# Appropriate and timely antibiotic administration for neonatal sepsis in Mesoamérica

**DOI:** 10.1136/bmjgh-2017-000650

**Published:** 2018-05-24

**Authors:** Herbert C Duber, Emily A Hartford, Alexandra M Schaefer, Casey K Johanns, Danny V Colombara, Emma Iriarte, Erin B Palmisano, Diego Rios-Zertuche, Paola Zuniga-Brenes, Bernardo Hernández-Prado, Ali H Mokdad

**Affiliations:** 1 Institute for Health Metrics and Evaluation, University of Washington, Seattle, Washington, USA; 2 Department of Emergency Medicine, University of Washington, Seattle, Washington, USA; 3 Department of Pediatrics, University of Washington, Seattle Children’s Hospital, Seattle, Washington, USA; 4 Salud Mesoamérica Initiative/Inter-American Development Bank, Panama City, Panama

**Keywords:** health systems, child health, health systems evaluation, paediatrics

## Abstract

Neonatal sepsis is a leading cause of mortality among children under-5 in Latin America. The Salud Mesoamérica Initiative (SMI), a multicountry results-based aid programme, was designed to improve maternal, newborn and child health in impoverished communities in Mesoamérica. This study examines the delivery of timely and appropriate antibiotics for neonatal sepsis among facilities participating in the SMI project. A multifaceted health facility survey was implemented at SMI inception and approximately 18 months later as a follow-up. A random sample of medical records from neonates diagnosed with sepsis was reviewed, and data regarding antibiotic administration were extracted. In this paper, we present the percentage of patients who received timely (within 2 hours) and appropriate antibiotics. Multilevel logistic regression was used to assess for potential facility-level determinants of timely and appropriate antibiotic treatment. Among 821 neonates diagnosed with sepsis in 63 facilities, 61.8% received an appropriate antibiotic regimen, most commonly ampicillin plus an aminoglycoside. Within 2 hours of presentation, 32.3% received any antibiotic and only 26.6% received an appropriate regimen within that time. Antibiotic availability improved over the course of the SMI project, increasing from 27.5% at baseline to 64.0% at follow-up, and it was highly correlated with timely and appropriate antibiotic administration (adjusted OR=5.36, 95% CI 2.85 to 10.08). However, we also found a decline in the percentage of neonates documented to have received appropriate antibiotics (74.4% vs 51.1%). Our study demonstrated early success of the SMI project through improvements in the availability of appropriate antibiotic regimens for neonatal sepsis. At the same time, overall rates of timely and appropriate antibiotic administration remain low, and the next phase of the initiative will need to address other barriers to the provision of life-saving antibiotic treatment for neonatal sepsis.

**Key questions**

**What is already known?**

- Neonatal sepsis is a leading cause of neonatal mortality worldwide and requires early recognition and treatment with appropriate empiric antibiotics.

**What are the new findings?**

- During implementation of the Salud Mesoamérica Initiative, improvement of facilities and provision of supplies was associated with improved delivery of antibiotics, but timely and appropriate treatment remained a challenge in neonatal sepsis.

**What do the new findings imply?**

- Ongoing quality improvement work and monitoring of patient outcomes is needed to improve treatment of neonatal sepsis.

## Introduction

The Millennium Development Goals set an ambitious target of reducing child mortality by two-thirds from 1990 to 2015. Although that goal has not been realised, under-five mortality rates have decreased globally between 2000 and 2016, from 69.4 per 1000 livebirths (67.2–71.8) to 38.4 per 1000 livebirths (34.5–43.1) since 2000.^1^ However, reductions in childhood mortality have not been evenly distributed. In particular, neonatal deaths declined at a significantly slower pace, with an estimated 2.2 million deaths within 28 days of birth in 2016.^1^

A common and often treatable condition, neonatal sepsis is the eighth leading cause of under-five mortality worldwide, accounting for more than 240 000 deaths globally in 2016.^2^ Despite being such a common cause of death, little progress has been made in reducing neonatal sepsis mortality between 2005 and 2015.^3^ In Latin America and the Caribbean (LAC), neonatal sepsis resulted in nearly 14 500 deaths in 2016, approximately one out of five neonatal deaths, and was the fifth leading cause of mortality and disability-adjusted life years among children under 5 years of age in the LAC region.^2^

Although the literature on managing neonatal sepsis is very limited outside of high-income settings, the provision of timely fluid resuscitation and antibiotic therapy are likely to portend significant benefits.^4–7^ Numerous studies throughout the age spectrum and across a wide array of high-income countries have demonstrated that rapid fluid resuscitation and antibiotic administration result in decreased mortality for patients presenting with severe sepsis and septic shock.^8^ In addition, providing timely and appropriate empiric broad-spectrum antibiotics is likely to be key in the neonatal setting where pathogens vary based on age. As a result, guidelines clearly reflect the need for providers to administer timely and appropriate antibiotics for neonates presenting with sepsis.^9 10^

In low-resource settings, timely empiric antibiotic administration depends on a number of factors which may prove challenging. These include both demand-side barriers, such as access to care and caregiver recognition of illness, and supply-side barriers, such as provider identification of sepsis and availability of appropriate antibiotics.^11–13^ In this study, we use data from the Salud Mesoamérica Initiative (SMI), a results-based funding programme that seeks to improve maternal, newborn and child health among some of the poorest populations between Southern Mexico and Panama, to examine treatment of neonatal sepsis in the LAC region. Specifically, we analyse the delivery of appropriate and timely antibiotic treatment in cases of neonatal sepsis across five Central American countries, focusing on changes over time and potential determinants of improved processes of care.

## Methods

### Study setting and design

A baseline measurement for SMI was conducted at select health facilities across Mesoamérica between 2011 and 2013. A follow-up measurement was conducted approximately 18 months after the initial country visit. In some cases, the same health facilities were visited at both baseline and follow-up, while in other cases facilities differed due to local conditions. Health facilities, the primary unit for this analysis, were grouped according to three levels of essential obstetric and neonatal care: ambulatory, basic and complete. Ambulatory facilities (excluded from this analysis) provide outpatient care, basic facilities are able to attend uncomplicated deliveries and provide immediate emergency obstetric and neonatal care, and complete facilities have the additional capacity to attend complicated deliveries and basic surgical services. A detailed overview of the SMI evaluation, including health facility selection, has been published elsewhere.^14^

The health facility survey included three main components: an interview questionnaire, an observation checklist and medical record reviews (MRR). In the interview questionnaire, the facility director was interviewed to capture information on general facility characteristics, infrastructure, human resources, consumables, logistics and facility-level processes. An observation checklist was used to record availability and functionality of essential equipment and supplies, including pharmaceuticals, on the day of the visit. Research assistants also reviewed administrative records of pharmaceutical stocks, capturing drug stock-outs occurring in the 3 months prior to the survey. Finally, in five countries (Belize, Guatemala, Honduras, Mexico and Nicaragua), MRRs were used to capture retrospective data on record-keeping and treatment practices for cases of maternal and neonatal complications, uncomplicated deliveries, antenatal and postpartum care, and child care. Neonatal complications included neonatal sepsis, low birth weight, prematurity and/or asphyxia. Medical records from these five countries with a recorded diagnosis of neonatal sepsis (reviewed at the time of data extraction by a medical professional) were included in our analysis.

In general, medical records within the 2-year period prior to the date of the health facility visit where one of the four neonatal complications of interest was diagnosed, were eligible for selection. Sampling quotas for each record type varied by country, round and facility. Sample size was largely determined based on practical considerations such as number of facilities to be visited within a given round, and the total number of anticipated MRRs to be performed across diseases of interest at a given facility. Where electronic discharge registries were available, records corresponding to the conditions of interest were randomly selected. Research assistants then sought out the selected medical records, and prespecified data elements were manually recorded. In the absence of electronic registries, records were sampled by hand using a systematic sampling technique to meet the quota for records within each facility. The systematic procedure encompassed estimating the number of cases for the desired condition in any given week, which was the sampling interval, and selecting a random week as the starting point for medical record selection. Records for the desired condition were included in the sample if they were directly selected or within two medical records before or two after the selected case. This procedure was meant to include a sample of medical records for the entire 2-year time frame considered by the indicator. When the target sample size was equal to or smaller than the total number of cases available, all medical records were selected.

In cases of neonatal sepsis, information was collected on time of health facility presentation, diagnostic tests performed on the neonate, name of antibiotic(s) received and time of antibiotic administration. In some cases where age was not documented, a medical professional confirmed cases of neonatal sepsis at the time of data extraction. All data were captured on laptop computers using an electronic data collection tool.

### Data analysis

This analysis only focuses on those MRRs where a diagnosis of neonatal sepsis was recorded. Using data extracted from MRRs at baseline and follow-up, we assessed whether the documented antibiotic coverage was appropriate for a presumed diagnosis of neonatal sepsis. Appropriate empiric antibiotic regimens were determined based on national and international guidelines, and supplemented by expert opinion where necessary.^9 10 15–18^ Time from presentation to antibiotic administration was calculated directly from times recorded in the medical record. Antibiotics administration was considered to be timely if any antibiotic was initiated within 2 hours of presentation. Although no guidelines currently exist, there is consensus that antibiotics should be initiated as soon as possible in cases of suspected neonatal sepsis, and 2 hours has been used as a quality improvement metric in other settings.^19^

All findings related to the delivery of appropriate and timely antibiotic administration are presented descriptively. In addition, we used multivariable mixed effects logistic regression models, accounting for clustering of observations at the facility level, to identify potential determinants of delivering timely and appropriate antibiotics. Characteristics included as covariates in this model included facility type (basic vs complete); relevant training within the past 12 months; paediatrician on staff; timing of data collection (baseline vs follow-up); country; and the availability of appropriate antibiotics at the facility during direct observation. Relevant trainings include integrated management of childhood illnesses (IMCI, or AIEPI by the Spanish acronym) or management of neonatal complications (prematurity, low birth weight, sepsis and asphyxia). All facilities were asked about an IMCI/AIEPI training at baseline and follow-up. However, information regarding additional dedicated training on the management of neonatal complications was not collected in Guatemala, Honduras or Nicaragua at follow-up. Two sensitivity analyses, one using only facilities that were included in both the baseline and follow-up periods, and another excluding basic facilities, were also performed. Stata V.13.1 (StataCorp 2013, College Station, TX) was used for all analyses.

### Ethical considerations

The study received institutional review board approval from the University of Washington, partnering data collection agencies and the Ministry of Health in each country. Informed consent was obtained from each health facility administrator prior to data collection, and medical records were anonymised during the data extraction process.

## Results

A total of 1923 neonatal MRRs were extracted, of which 831 cases had a diagnosis of neonatal sepsis. Ten patients left against medical advice prior to antibiotic administration and were excluded from this analysis, resulting in a final count of 821 records. Table 1 presents the distribution of cases by facility type (complete vs basic) and country at baseline and follow-up evaluation. 43.7% of cases were female, 53.0% were male and 3.3% did not have a sex documented. 10.2% of records did not record an age; of those that recorded an age, 81.6% presented in the early neonatal period (between 0 and 7 days of age).

**Table 1:** MRRs extracted by country and facility type

Country	Baseline (n=375)		Follow-up (n=446)		Total (n=821)
	Basic (n=60)	Complete (n=315)	Basic (n=139)	Complete (n=307)	
Belize	1 (1.7%)	32 (10.2%)	6 (4.3%)	21 (6.8%)	60 (7.3%)
Guatemala	0 (0.0%)	61 (19.4%)	17 (12.2%)	48 (15.6%)	126 (15.4%)
Honduras	0 (0.0%)	137 (43.5%)	20 (14.4%)	113 (36.8%)	270 (32.9%)
Mexico	20 (33.3%)	62 (19.7%)	22 (15.8%)	89 (29.0%)	193 (23.5%)
Nicaragua	39 (65.0%)	23 (7.3%)	74 (53.2%)	36 (11.7%)	172 (21.0%)

MRR, medical record review.

A total of 63 unique facilities were visited, although only 27 were included in both the baseline and follow-up samples. Seventeen facilities were in Mexico, 15 each in Honduras and Nicaragua, 12 in Guatemala and only 4 in Belize. A total of 40 healthcare facilities were visited during the baseline study period and 50 at the 12-month follow-up visit (table 2). Just over half (33) of the unique facilities were classified as basic, although the distribution of basic versus complete facilities differed at baseline and 12-month follow-up (72.5% vs 44.0% complete facilities). 82.5% of facilities had a paediatrician on staff at the time of the baseline survey and 76.0% at the time of follow-up. IMCI, AIEPI or management of neonatal complications training occurred at 75.0% and 84.0% of facilities within the 12-month period preceding baseline and follow-up, respectively. Antibiotics were significantly more available at the time of the follow-up survey (27.5% vs 64.0%).

**Table 2:** Facility characteristics at baseline and follow-up

	Baseline (n=40)	Follow-up (n=50)
Complete-level facility	29 (72.5%)	22 (44.0%)
Country		
Belize	3 (7.5%)	4 (8.0%)
Guatemala	5 (12.5%)	11 (22.0%)
Honduras	10 (25.0%)	11 (22.0%)
Mexico	16 (40.0%)	11 (22.0%)
Nicaragua	6 (15.0%)	13 (26.0%)
Paediatrician on staff	33 (82.5%)	38 (76.0%)
Relevant training* within prior 12 months	30 (75.0%)	42 (84.0%)
Availability of appropriate antibiotic combination	11 (27.5%)	32 (64.0%)

*Relevant training includes IMCI/AIEPI/management of neonatal complications.

IMCI, integrated management of childhood illnesses.

### Appropriate and timely antibiotic administration

**Figure 1:**
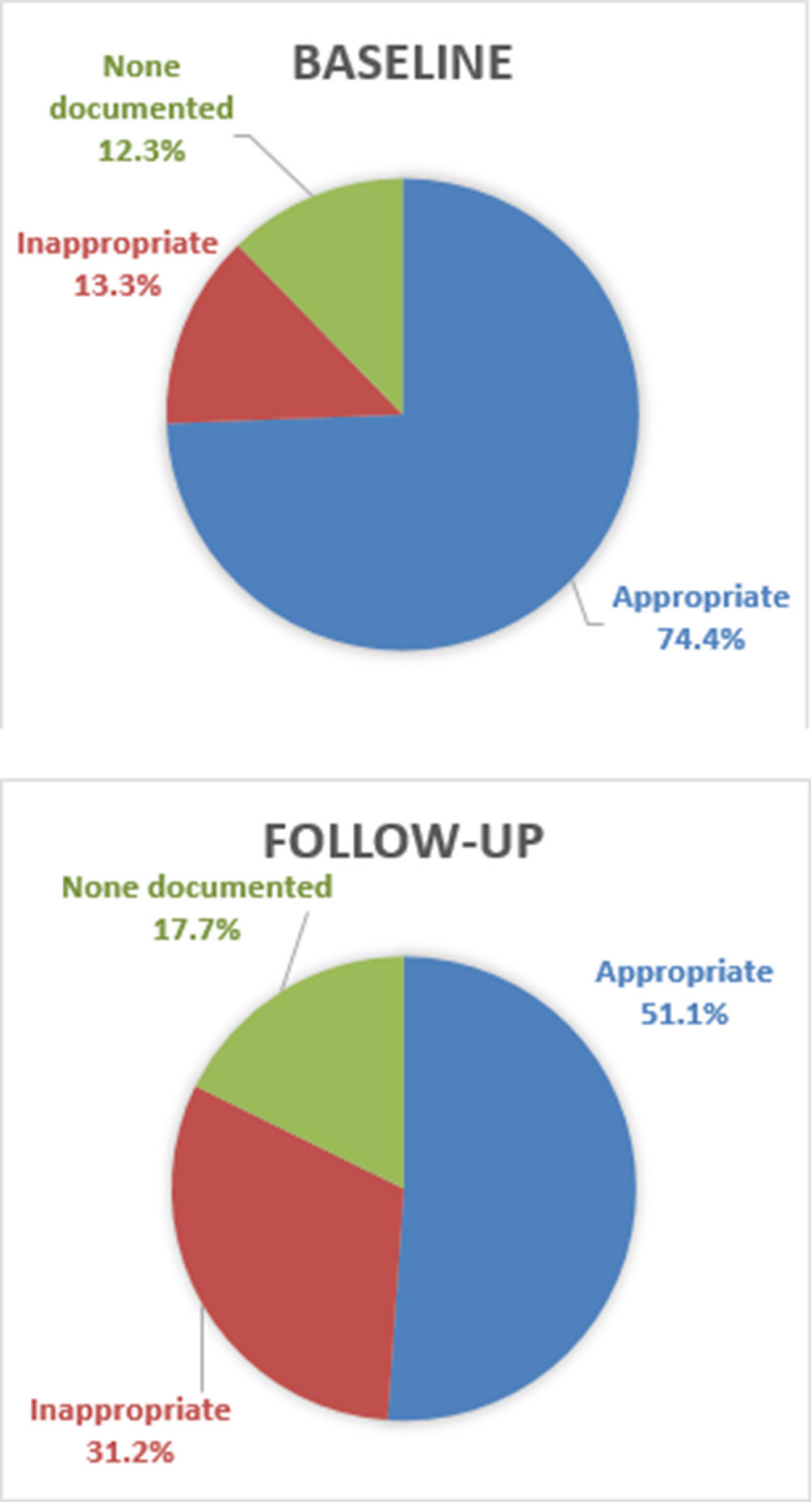
Administration of appropriate antibiotics at baselines and follow-up.

Overall, we found that an appropriate empiric antibiotic regimen was documented in 61.8% of MRRs with a diagnosis of neonatal sepsis. 1.1% received an inappropriate combination, 21.9% were administered only one antibiotic and no antibiotics were documented in 15.2% of cases. Comparing baseline and follow-up, we found that a smaller percentage of patients received appropriate antibiotics at follow-up (74.4% vs 51.1%), while the number that received any antibiotic also declined slightly from 87.7% to 82.3% (figure 1).

The most common appropriate antibiotic regimen, administered in 83.8% of cases where an appropriate regimen was provided, was ampicillin and an aminoglycoside. Crystalline penicillin and amikacin was the next most common empiric antibiotic regimen, administered in 5.0% of cases where appropriate antibiotics were provided. Ampicillin was by far the most commonly used monotherapy, occurring in 60.6% of cases where a single antibiotic was documented.

**Figure 2:**
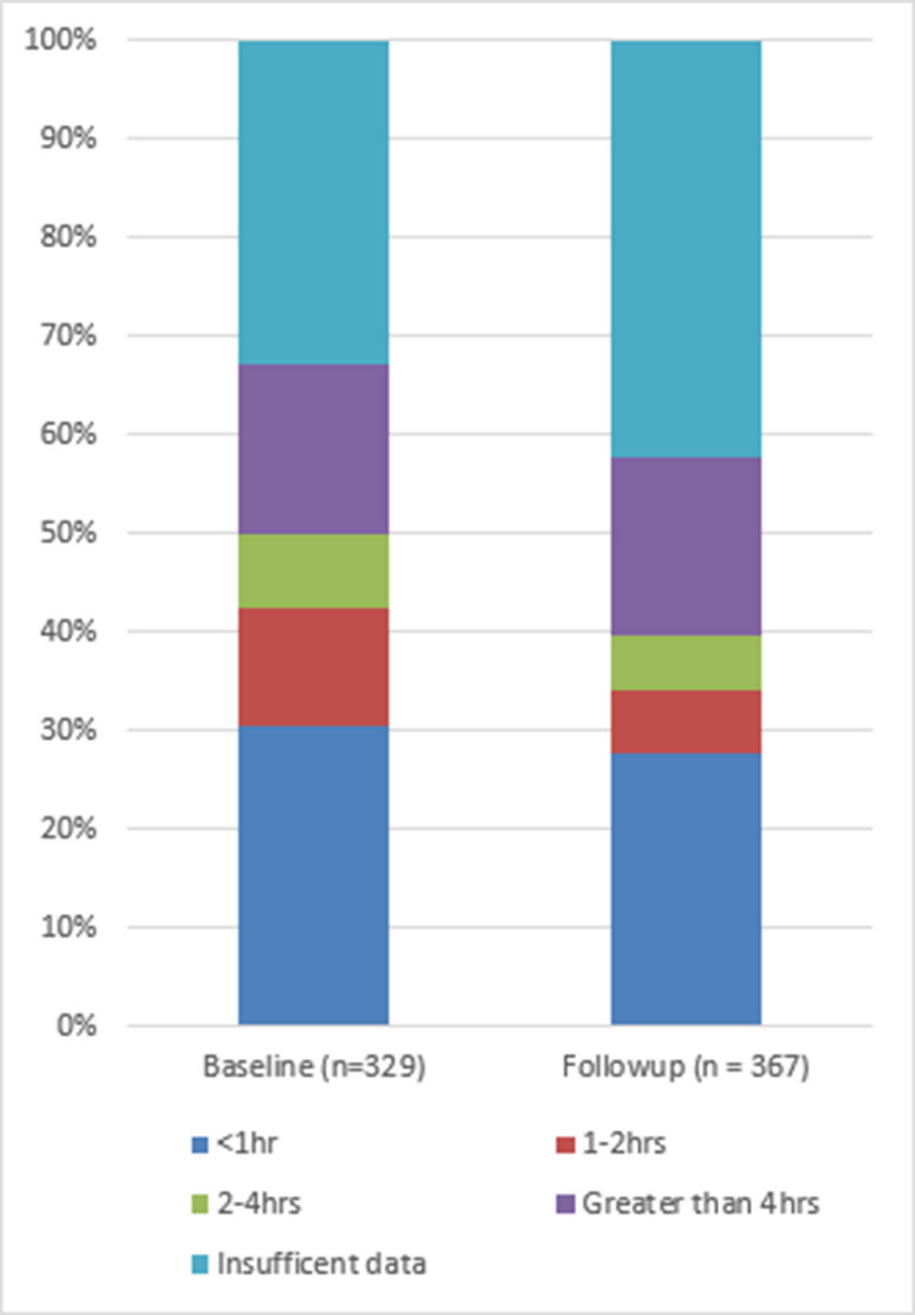
Time to antibiotic administration.

**Figure 3:**
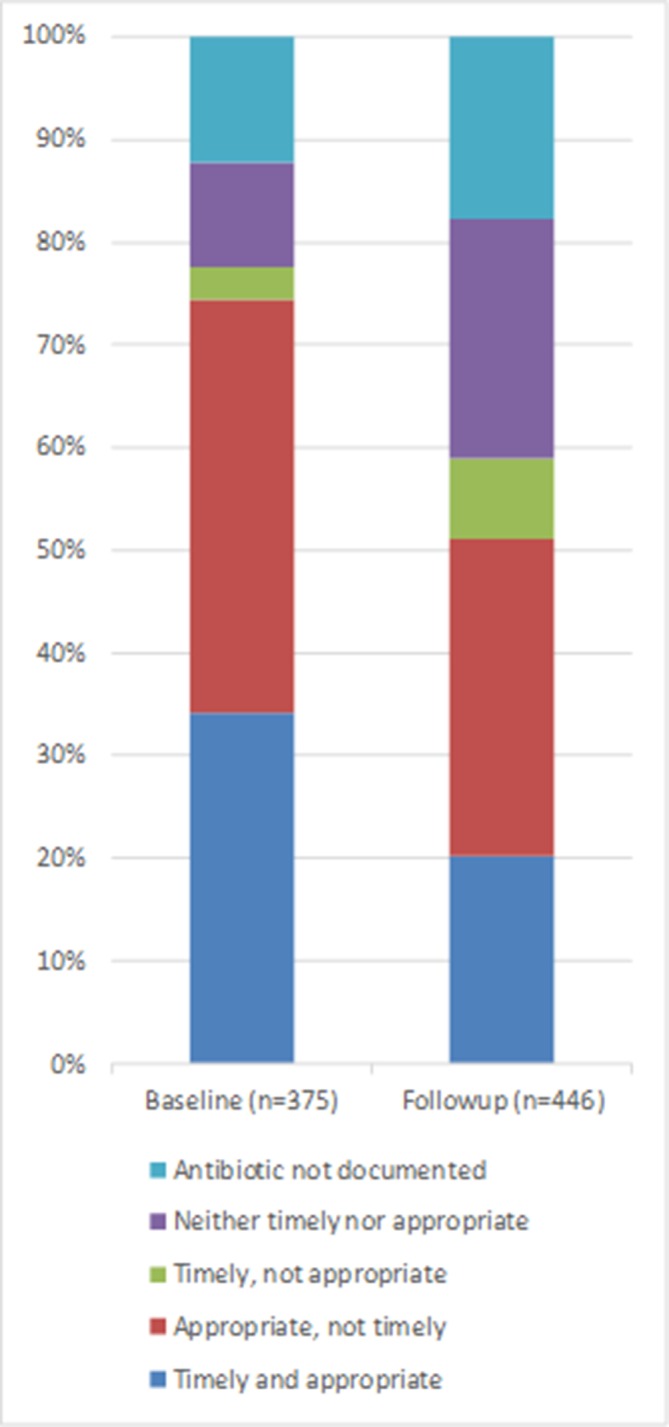
Administration of appropriate and timely antibiotics.

Neonates were documented to have received any antibiotic within 1 hour of presentation in 26.7% of cases at baseline and 22.9% at follow-up (figure 2). Within 2 hours, the percentage receiving any antibiotic increased to 37.3% and 28.0% at baseline and follow-up, respectively. MRRs documenting receipt of an appropriate regimen within 2 hours of presentation decreased from 34.1% at baseline to 20.2% at follow-up (figure 3).

**Figure 4:**
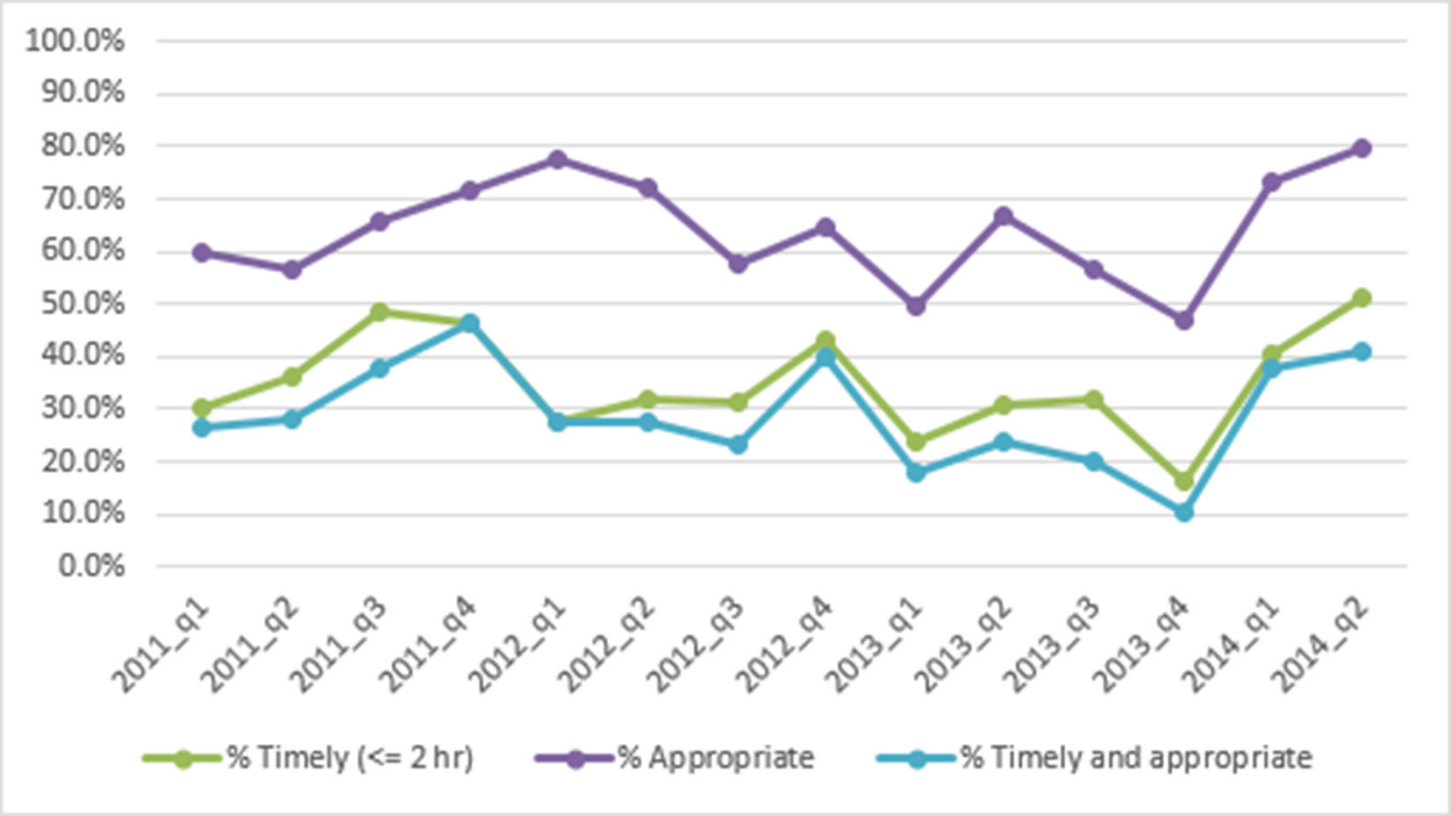
Timely and appropriate antibiotics by quarter.

Documentation of an appropriate antibiotic regimen initiated within 2 hours of diagnosis occurred in 26.6% of all cases of neonatal sepsis. Figure 4 allows for a more granular view of appropriate, timely (<2 hours), and both appropriate and timely antibiotic administration over the course of the project. Although data were collected at two discrete time periods, baseline and follow-up, medical records were drawn from the 2 years prior to date of data collection as described above. Descriptively, we see no definitive trend in any of the three outcome metrics over the study period.

We also note that several covariates were significantly associated with the administration of appropriate and timely antibiotic therapy (table 3). Antibiotic availability at the facility was highly correlated with appropriate and timely antibiotic administration (OR 5.36 (2.85, 10.08)). In addition, we found substantial country-level variability, although CIs were large and overlapping. Interestingly, we also note that neonates with sepsis were less likely to receive appropriate and timely antibiotics at the follow-up (OR 0.32 (0.15, 0.68)). All of these relationships held steady in sensitivity analyses (table 3).

**Table 3:** Association between facility characteristics and the provision of timely and appropriate empiric antibiotics for neonatal sepsis (adjusted OR (95% CI))

	All cases (n=821)	Cases seen at facilities included at both baseline and follow-up (n=616)	Cases seen at complete-level facilities only (n=622)
Complete-level facility	0.68 (0.23 to 2.02)	0.26 (0.05 to 1.29)	Omitted
Country			
Belize	0.99 (0.57 to 1.73)	0.90 (0.46 to 1.79)	0.94 (0.57 to 1.55)
Guatemala	*0.39 (0.20 to 0.77)*	*0.42 (0.19 to 0.94)*	*0.37 (0.18 to 0.76)*
Honduras	Ref	Ref	Ref
Mexico	*0.13 (0.06 to 0.30)*	*0.11 (0.04 to 0.31)*	*0.08 (0.02 to 0.26)*
Nicaragua	*0.18 (0.05 to 0.60)*	*0.05 (0.01 to 0.29)*	*0.04 (0.00 to 0.35)*
Paediatrician on staff	3.19 (0.40 to 25.76)	1.16 (0.21 to 6.56)	0.54 (0.17 to 1.78)
Relevant training* within prior 12 months	0.57 (0.26 to 1.24)	0.51 (0.19 to 1.34)	0.43 (0.18 to 1.06)
Availability of appropriate antibiotic combination	*5.36 (2.85 to 10.08)*	*7.40 (3.01 to 18.15)*	*6.95 (2.66 to 18.16)*
18-month follow-up	*0.32 (0.15 to 0.68)*	*0.27 (0.09 to 0.81)*	*0.26 (0.09 to 0.78)*

*Relevant training includes IMCI/AIEPI/management of neonatal complications.

IMCI, integrated management of childhood illnesses.

## Discussion

This is the first study to examine the administration of timely and appropriate antibiotics for neonatal sepsis across some of the poorest areas of Mesoamérica. This analysis revealed that among health facilities serving impoverished communities there were improvements in the availability of antibiotics over the first phase of the SMI. Additionally, we found that antibiotic availability was highly associated with the provision of appropriate antibiotic therapy for neonatal sepsis. However, we also found significant opportunities to improve the delivery of appropriate and timely empiric antibiotic treatment for neonates diagnosed with sepsis. These findings are of great importance given the persistent burden of neonatal mortality in the region, and provide evidence for action by adopting lessons learnt.

From an implementation perspective, the first phase of the SMI (from baseline to follow-up) was intended to focus on creating the conditions to improve quality and coverage during subsequent phases (ie, structural quality). Interventions included: strengthening supply chains and management practices, updating protocols, strengthening the organisation of the health network, and designing future work which will focus on coverage and quality of care. However, nearly one-third of facilities still lacked the necessary antibiotics to treat neonatal sepsis. The later phases of SMI, some of which are currently in progress, focus on process and healthcare quality improvement. Continued emphasis on providing the tools necessary to improve care will be critical, and a root cause analysis to assess potential reasons for our finding would be a reasonable next step.

Interestingly, we find that the gap in providing timely antibiotic treatment was not solely limited by the availability of appropriate antibiotics. In fact, timely treatment with any antibiotic occurred in only one-third of cases, suggesting that medication availability alone does not translate into quality of care. Furthermore, neither the availability of a paediatrician on staff nor commonly used trainings such as IMCI/AIEPI were associated with improvement in timely and appropriate antibiotic administration. The finding that a paediatrician on staff does not improve quality of care is not completely surprising as this does not equate to having a paediatrician present at the time of evaluation, and/or on-call at the time of case presentation. Additionally, the time constraints associated with rapid appropriate antibiotic treatment require that non-paediatrician staff quickly assess neonates and deliver the necessary antibiotic therapy without consultation. Further action based on process improvement, team-building and empowering non-physician and non-specialty healthcare workers at rural facilities will be an important step in improving neonatal sepsis treatment.

Innovative approaches and programmes leading to significant reductions in time to antibiotic therapy have been described in high-income settings, but their adaptation to low/middle-income countries has rarely been assessed.^20^ In a recently published quality improvement project in rural Bangladesh, the authors noted a large improvement, from 45% to 75%, in choice of appropriate first-line antibiotics.^21^ The Bangladesh project incorporated education for rapid neonatal assessment and process improvements for timely care. This suggests Ministries of Health will need to focus on quality improvement, with an emphasis on processes of care for early diagnosis and treatment of neonatal sepsis.

As neonatal deaths continue to represent a growing proportion of overall child mortality, addressing the quality of care for neonatal sepsis and other causes of death for neonates who reach health facilities will be increasingly important. A recent analysis of serious bacterial infections in neonates across Latin America, India and sub-Saharan Africa found comparatively high incidence of neonatal sepsis as well as case fatality rates.^22^ Despite significant country-level variation, similar to that described here, these findings reinforce the need to focus on neonatal health.

This call to action is emphasised by the *Every Newborn* Action Plan, which builds on the IMCI programme widely credited for improving healthcare worker performance, process-oriented quality of care and intermediate outcomes, such as improved nutritional status.^23–25^ Launched in 2014 by the WHO and Unicef, the Action Plan identifies a research and coordination strategy to move towards the elimination of stillbirths and neonatal deaths.^26^ One of the programmes’ key research priorities is the development of strategies to improve the identification and management of neonatal infections. Furthermore, the Action Plan specifically addresses the need to close the gap in quality of care for newborns, most notably in the critical first week of life when neonates are at highest risk for infection and other complications. Implementation of this agenda will require increased financial investment, political buy-in and overcoming myths regarding futility of investment in interventions focused on improving survival in the neonatal period.^27–29^

Our findings should be considered within the context of certain limitations. First, we include MRRs where a diagnosis of neonatal sepsis was made, but do not define criteria for this diagnosis. We do this because the definition of neonatal sepsis is not without controversy, and the availability of data within the medical record to make a diagnosis would often be insufficient—either due to lack of definitive testing or documentation. There were also likely some infants where the provider suspected sepsis and opted to transfer the child to another facility for diagnosis and management without initiating treatment.^30^ However, it is unlikely that systematic differences in diagnosis exist across facilities included in this study. Second, as in many retrospective observational chart reviews, our analysis is limited by documentation. It is possible that appropriate and/or timely antibiotics were administered in cases where they were not documented. However, even in cases where there were sufficient data to assess timeliness and an antibiotic was documented, less than half of these patients received appropriate antibiotics within 2 hours. In the facilities of interest, there are few other options to measure quality improvement in a cost-effective way, and future education should include an emphasis on the critical nature of documentation. Finally, it is possible that we did not account for key unobserved covariates in modelling the association between facility characteristics and the provision of timely and appropriate antibiotics.

## Conclusion

Our study demonstrated improvements in the availability of appropriate antibiotic regimens for neonatal sepsis in the early phase of the SMI. This is promising and calls for increased efforts to sustain this success. Simultaneously, overall rates of timely and appropriate antibiotic administration remain low, and the next phase of the initiative will need to address some of the challenges identified. Increased focus on quality improvement by Ministries of Health across Mesoamérica is needed; increasing the availability of key inputs and improving processes of care for the diagnosis and management of neonatal sepsis. Our findings have many implications on the health systems of the region and will guide efforts to address neonatal mortality in the region.
